# Transcriptomic signatures across a critical sedimentation threshold in a major reef-building coral

**DOI:** 10.3389/fphys.2024.1303681

**Published:** 2024-06-11

**Authors:** Colin Lock, Melissa M. Gabriel, Bastian Bentlage

**Affiliations:** Marine Laboratory, University of Guam, Mangilao, GU, United States

**Keywords:** apoptosis, cell adhesion, immune response, metabolism, gene expression, stress, Symbiodiniaceae, Porites

## Abstract

Sedimentation is a major cause of global near-shore coral reef decline. Although the negative impacts of sedimentation on coral reef community composition have been well-documented, the effects of sedimentation on coral metabolism *in situ* have received comparatively little attention. Using transcriptomics, we identified gene expression patterns changing across a previously defined sedimentation threshold that was deemed critical due to changes in coral cover and community composition. We identified genes, pathways, and molecular processes associated with this transition that may allow corals, such as *Porites lobata*, to tolerate chronic, severe sedimentation and persist in turbid environments. Alternative energy generation pathways may help *P. lobata* maintain a persistent stress response to survive when the availability of light and oxygen is diminished. We found evidence for the expression of genes linked to increased environmental sensing and cellular communication that likely allow *P. lobata* to efficiently respond to sedimentation stress and associated pathogen challenges. Cell damage increases under stress; consequently, we found apoptosis pathways over-represented under severe sedimentation, a likely consequence of damaged cell removal to maintain colony integrity. The results presented here provide a framework for the response of *P. lobata* to sedimentation stress under field conditions. Testing this framework and its related hypotheses using multi-omics approaches can deepen our understanding of the metabolic plasticity and acclimation potential of corals to sedimentation and their resilience in turbid reef systems.

## Introduction

Coral reefs play important cultural, ecological, and economic roles in Guam and throughout the Northern Mariana Islands (Burdick et al., 2008). Reefs recycle nutrients, provide a habitat for marine organisms, and prevent coastline erosion from strong storms and wave action (Hughes et al., 2003). Stony corals (Scleractinia) are complex holobionts, with approximately half of the more than 1,600 described species hosting an endosymbiotic assemblage of dinoflagellate algae (Symbiodiniaceae) that are central to metabolic homeostasis through nutritional mutualism (Gault et al., 2021). However, climate change and other anthropogenic stressors threaten this mutualistic relationship and, in turn, coral reefs (National Academies of Sciences, Engineering, and Medicine, 2019). Upland erosion caused by human activities such as wild-land arson, deforestation, construction and development, and recreational off-roading has increased turbidity and sedimentation in Guam’s watersheds, exerting significant stress on near-shore coral reef ecosystems (Reynolds et al., 2014; Minton et al., 2022). The destruction of wetlands and streams by coastal development has further increased sedimentation impacts on near-shore reefs in Guam (Scheman et al., 2002; Wolanski et al., 2003; Minton et al., 2022). The increasing erosion of soils and the resulting near-shore sedimentation are widespread global problems, leading to a decline in diversity and ecosystem services provided by coral reefs (Rogers and Ramos-Scharrón, 2022).

Suspended sediment particles increase turbidity and form aggregations that are deposited on corals through sedimentation, negatively impacting coral metabolism (Weber et al., 2012; Sheridan et al., 2014; Bollati et al., 2021; Tuttle and Donahue, 2022). Fine sediments and decaying organic matter deposited in near-shore reef systems deplete the available oxygen, which decreases pH and increases the oxygen demand of corals, resulting in oxidative stress (Erftemeijer et al., 2012; Flores et al., 2012; Tuttle and Donahue, 2022). To survive under such conditions, corals employ mitigation processes, such as increased mucus production and ciliary movement to shed the deposited sediment (Weber et al., 2012; Bessell-Browne et al., 2017), exerting high energetic costs that can lead to a decrease or cessation of other metabolic functions (Riegl and Branch, 1995; Anthony and Larcombe, 2000; Tuttle and Donahue, 2022). Sedimentation triggers coral immune responses that further deplete energy stores (Sheridan et al., 2014) and increase disease susceptibility (Sheppard et al., 2009). Increased turbidity caused by suspended sediments results in a decrease in the photosynthetic activity of coral-associated Symbiodiniaceae (Fabricius, 2005; Jones et al., 2020; Philipp and Fabricius, 2003). Bacteria (Franco et al., 2020) and anthropogenic chemicals (Ishibashi and Takeuchi, 2023) associated with runoff and sedimentation present further challenges beyond the physical removal of particulates from coral polyps. Critical thresholds for sedimentation at which coral mortality drastically increases range from as low as 10 mg cm^-2^ day^-1^ to as high as 300 mg cm^-2^ day^-1^, depending on the coral species impacted, reef location, types of sediments deposited, and length of exposure to sedimentation (Erftemeijer et al., 2012; Jones et al., 2020; Tuttle and Donahue, 2022). Massive *Porites* spp., such as *Porites lobata*, are one of the few coral species groups known for their persistence under moderate-to-severe sedimentation (Rogers, 1990; Golbuu et al., 2011). Many *Porites* species have been observed to produce thick mucus envelopes that effectively help with the removal of sediment from tissue, and mucus thickness appears to be correlated with increasing sediment load (Bessell-Browne et al., 2017). Documented responses of *P. lobata* to sedimentation range from mortality caused by sedimentation rates as low as 30 mg cm^-2^ day^-1^ (Hodgson, 1990) to bleaching but no apparent mortality under severe sedimentation of 200 mg cm^-2^ day^-1^ for 6–8 days (Stafford-Smith, 1993). Large discrepancies in the reported sedimentation tolerances of *P. lobata* may suggest physiological plasticity within this species or represent uncertainties in the identification of massive *Porites* spp. and species-specific differences in acclimation potential. Regardless, the persistence of massive *Porites* spp. in environments affected by moderate-to-severe sedimentation represents unique opportunities for identifying the mechanisms of acclimation to sedimentation.

**FIGURE 1:**
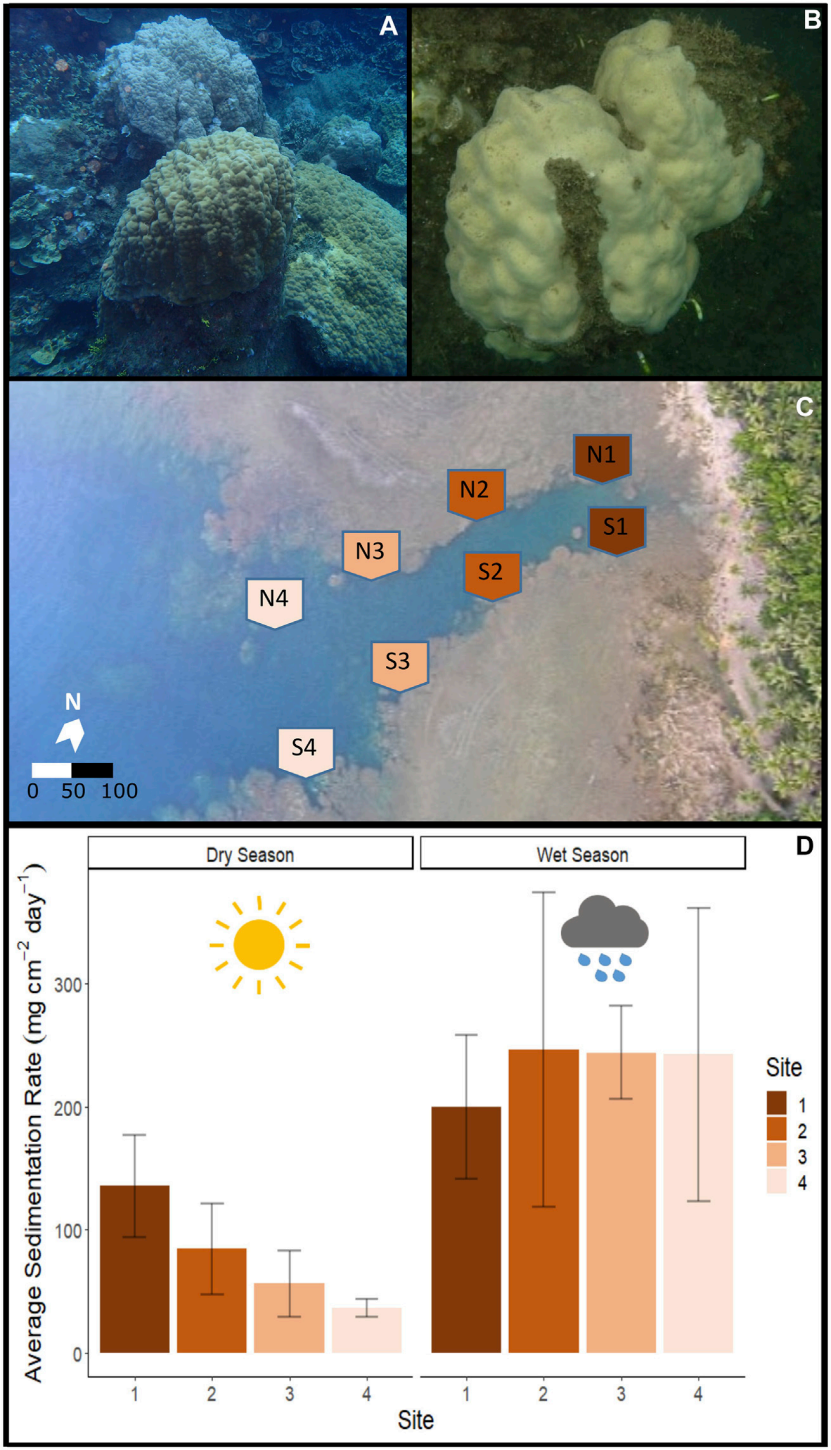
Fouha Bay (Guam) sampling locations and sedimentation rates. **(A)** *Porites lobata* colony sampled from the outer zone (N4) that shows minimal sediment accumulation (images taken during the dry season). **(B)** *P. lobata* colony sampled from the inner location (S1) that shows heavy sediment accumulation. **(C)** Aerial view of Fouha Bay and sampling sites. **(D)** Average sedimentation rate across sampling sites (N/S 1–4) during the dry and wet seasons.

Transcriptome-level characterization of gene expression in corals provides insights into complex metabolic processes, which deepens our understanding of how corals respond to stressors, such as sedimentation, and, ultimately, their future survival (Barshis et al., 2013; Barshis et al., 2014). This approach has been used successfully to lay the foundation for identifying the molecular mechanisms that allow certain coral species to survive or thrive in marginal habitats (Barshis et al., 2014). Understanding the thresholds of sedimentation tolerance in corals may inform decision-making processes for coral reef conservation and restoration (Burdick et al., 2008; Barshis et al., 2014; Hughes et al., 2017; Tuttle and Donahue, 2022). Recent experimental work investigated coral gene expression in response to acute sedimentation under laboratory conditions and identified genes associated with energy metabolism and immune responses (Bollati et al., 2021). Under laboratory conditions, coral responses to sedimentation are generally tested by smothering corals under sediments for short periods of time (Bollati et al., 2021; Tuttle and Donahue, 2022). Considering that sedimentation stress under ecologically relevant conditions in the field may be more complex than laboratory experiments simulate, we used transcriptome data from field-collected corals to test whether the genes and pathways identified by Bollati et al. (2021) are differentially expressed under field conditions in a reef system impacted by sedimentation. Specifically, we employed transcriptomics to examine the gene expression profiles of *P. lobata* colonies tagged *in situ* for repeat sampling (Figure 1A, B) to understand the metabolic response of *P. lobata* and its endosymbiotic Symbiodiniaceae community living in a habitat characterized by moderate-to-severe sedimentation. Our analyses identified significant changes in gene expression profiles across a previously identified critical threshold of sedimentation across which coral mortality increases and community composition changes significantly in Fouha Bay, southern Guam (Minton et al., 2022). The genes identified by our analyses are consistent with the coral response mechanisms to sedimentation identified previously under laboratory conditions and provide insights into coral acclimation mechanisms in turbid, sedimentation-impacted reef ecosystems that act on an ecologically relevant scale.

## Materials and methods

### Study site and environment

The Humåtak watershed in southern Guam (13°28′ N, 144°45′ E) is characterized by basaltic rock, steep slopes, and lateritic soils that readily erode during heavy rainfalls (Foster and Ballendorf, 2023). The La Sa Fu’a River and its 5 km^2^ catchment area belong to the Humåtak watershed that drains into Fouha Bay (Scheman et al., 2002), providing mean monthly freshwater discharges ranging from 0.3 m^3^ s^-1^ (March–May) to 2.9 m^3^ s^-1^ (August–October) (United States Geological Survey monitoring site 16809600 data averaged from 1953 to 2018; USGS, 2023), corresponding to dry- and wet-season rainfall patterns. Heavy rain events that occur primarily during the wet season and last, on average, 1–2 h transport sediment from eroding soils downstream into Fouha Bay, creating pulses of thick plumes that frequently exceed a suspended solid concentration (SSC) of 1,000 mg L^-1^ (Wolanski et al., 2003). Suspended solids flocculate and slowly precipitate as marine snow when reaching the bay, dissipating the plume over a time span of approximately 5 days (Wolanski et al., 2003). Large sedimentation events occur, on average, 10 times annually in Fouha Bay, with approximately 75% of sediments staying within the bay for an estimated residence time of 4.5 years (Wolanski et al., 2003).

Massive *Porites* of the *P. lobata*/*lutea* species complex (Figures 1A, B) represent one of the few coral species groups that persist in Fouha Bay along a gradient of sedimentation ranging from severe (sedimentation rate >50 mg cm^-2^ day^-1^) to moderate (sedimentation rate ∼10–∼50 mg cm^-2^ day^-1^) to light (sedimentation rate <10 mg cm^-2^ day^-1^) as distance from the river mouth increases (Minton et al., 2022). Sites close to the river mouth are further characterized by decreased light available for coral photosynthesis due to high turbidity and decreased salinity due to freshwater discharge (Fifer et al., 2022).

To evaluate previously established sedimentation dynamics across Fouha Bay (Rongo, 2004; Minton et al., 2022), sedimentation rates during the period of this study were estimated using a sediment trap deployed at eight sites established within the bay following the procedure used by Rongo (2004) (Figure 1C). We deployed U24 HOBO (Onset, Bourne, MA) data loggers to record water conductivity and temperature at each sampling site. Conductivity was converted to salinity using the Practical Salinity Scale 1978 (PSS-78), as implemented in HOBOware Pro of Onset. Sediment was collected over a time period of 31 days during the dry (2 May 2018 to 1 June 2018) and wet (21 September 2018 to 22 October 2018) seasons using vertical PVC traps (diameter of 5 cm and height of 50 cm), following the recommendations of Storlazzi et al. (2011). The collected sediments were processed following the protocol proposed by Ensminger (2016). Wet-strengthened 120-micron filters (Whatman, Little Chalfont, United Kingdom) were first rinsed with deionized water and dried for 24 h at 100°C; the weight of each dried filter was recorded. The collected sediment samples were rinsed with deionized water to remove salt and allowed to settle again for 24 h; the organisms contained in the sediment samples were removed. The cleaned samples were then filtered using the previously dried 120-micron filters using a vacuum pump, followed by the drying of sediments collected on filter paper at 100°C.

The drying filters and sediments were weighed repeatedly over a period of 2 days until the weights remained constant, indicating that the samples had dried completely, allowing for the calculation of the accumulated sediment. The total accumulated sediment for each sample was normalized to mg cm^-2^ day^-1^ by dividing it by the diameter of the PVC sediment trap and the number of days that trap had been deployed. A Shapiro–Wilk test was used to test normality in the sedimentation dataset, and then ANOVA was used to compare sedimentation rates between sites, seasons, and sides (using the AOV function in base R, version 4.2.3). Similarly, ANOVA was used to test for differences in the daily average temperature and salinity between all sites and seasons and the interaction of sites and seasons on the days of transcriptomic sampling (16 May 2018 and 3 October 2018 for dry and wet seasons, respectively).

### Coral tagging and identification

To identify the response of *P. lobata* and its Symbiodiniaceae endosymbionts (the experimental unit) in Fouha Bay, individual corals representing independent biological replicates (the unit of observation) were tagged at eight sites in Fouha Bay. At each of the eight sites in Fouha Bay (Figure 1C), at least three corals were tentatively identified in the field as *P. lobata* based on gross morphology and tagged for repeated sampling with cow tags that were zip-tied to adjacent reef pavements. Sediments and corals were sampled from the north and south sides of the channel into which the La Sa Fu’a River drains to capture the variation in sediment deposition and transcriptomic response, respectively. Coral colonies ranged in diameter from approximately 15 cm to 2 m, with colonies closer to the river mouth being smaller than more distant ones. Replicate colonies were chosen so that all colonies tagged were representative of the size classes observed at each site. Species were identified using a combination of corallite morphology and, given the difficulty of species-level identification of massive *Porites* species, DNA barcoding. Approximately 4 cm^2^ fragments from all 24 tagged colonies were collected from the center of the colony using a hammer/chisel and preserved in ethanol for DNA extraction.

DNA was extracted from each tagged colony using the GenCatch Genomic DNA Extraction Kit (Epoch Life Science, Sugar Land, TX) following the manufacturer’s protocol for tissue samples. Mitochondrial regions COX3-COX2 and ND5-tRNA-Trp-ATP8-COX1 were amplified with primer sets mt-16 and mt-20, respectively (Paz-García et al., 2016), in 25 μL reactions using 0.3 µM primers, 0.3 mM dNTP, 1× HiFi Fidelity Buffer, and 2.5 units of Taq (KAPA HiFi 1U). The thermocycler profile included an initial denaturation at 94°C for 120 s, followed by 30 cycles of 94°C for 30 s, 54°C for 30 s, and 72°C for 60 s, followed by a final extension at 72°C for 300 s. PCR products were sequenced using Sanger sequencing, and the resulting sequences were assembled using the overlap–layout–consensus algorithm implemented in Geneious Prime (Biomatters, Auckland, New Zealand). Following assembly, consensus sequences for each specimen were aligned to publicly available *Porites* spp. mitochondrial genomes using MAFFT v7.453. The maximum likelihood phylogeny was inferred from the concatenated, aligned regions using RAxML v8.2.12 (Stamatakis, 2014) under the GTRCAT model to allow for the efficient modeling of site heterogeneity across alignment regions that spanned multiple genes. The resulting phylogeny was rooted using *Porites fontanesii* following recent phylogenomic analyses (Terraneo et al., 2021); the robustness of the phylogeny was assessed using 1,000 non-parametric bootstrap replicates. Specimens grouped with *Porites lutea* were excluded from RNA sequencing, while specimens grouped with *P. lobata* were included in the set of specimens selected for gene expression analysis.

### RNA extraction, sequencing, and transcriptome assembly

Tagged colonies were sampled on 16 May 2018 during the dry season and on 3 October 2018 during the wet season, following the passing of two tropical depressions that led to significant rainfalls. A single fragment of each coral colony was sampled using a hammer/chisel and immediately preserved in RNAlater (Sigma-Aldrich, St. Louis, MO, United States), followed by storage at −80°C until RNA extraction. RNA from specimens identified as *P. lobata* (see section above) was extracted using the E.Z.N.A. Mollusc RNA Kit (Omega Bio-Tek, Norcross, GA, United States), following the manufacturer’s protocol. The extracted RNA was quantified using a Qubit Fluorometer (Life Technologies, Carlsbad, CA), and its integrity was verified using a bioanalyzer (Agilent Technologies, Santa Clara, CA), leading to the selection of 32 samples from different colonies for RNA sequencing. Sequencing libraries were constructed using NEBNext library kits (New England Biolabs, Ipswich, MA, United States) and sequenced on a NextSeq 550 Sequencer (Illumina, San Diego, CA, United States), generating 150-bp paired-end data. Adapter sequences and bases with a Phred-scaled quality score of less than 30 were removed using Trim Galore (Martin, 2011). The sequence data were deposited in GenBank of the NCBI (accession numbers are provided in Supplementary Table S1).

We employed *de novo* transcriptome assembly, following the best practices outlined by Grabherr et al. (2013). After the exclusion of two samples that yielded few sequencing reads (Supplementary Table S2), the sequencing data were combined and normalized using the Trinity v2.10.0 (Grabherr et al., 2013) *in silico* normalization function with maximum coverage set to 50 to reduce the computational requirements of the transcriptome assembly that used the default parameters of Trinity. After transcriptome assembly, TransDecoder v5.5.0 (Haas et al., 2013) was used to predict open reading frames (ORFs), followed by the removal of non-coding transcripts. Non-target contaminant sequences were identified and removed from the transcriptome assembly using the Perl script Alien Index (Ryan, 2014) with the approach outlined by Lock et al. (2022). Representative proteomes inferred from translated coding sequences (391,427 sequences) for each major bacterial clade (Schulz et al., 2017), fungi, *Stramenopiles*, poriferans, arthropods, molluscs, and annelids were obtained from GenBank of the NCBI and indexed in a non-target (alien) BLAST (Altschul et al., 1990) database; 57,401 coral and 72,664 Symbiodiniaceae proteome sequences were included as target sequences (file S1). Protein BLAST searches (e-value < 1e^−3^) using the ORFs predicted from our transcriptome assembly by TransDecoder as queries were run against the combined non-target (alien) and target (Cnidaria plus Symbiodiniaceae) protein databases to identify and remove likely contaminant sequences from our *de novo* assembled transcriptome using the Alien Index (Ryan, 2014). Target coral and Symbiodiniaceae ORFs were annotated based on the best BLAST hit (e-value < 1e-5) against a database containing 129,600 cnidarian (including corals and other cnidarians) and 44,114 Symbiodiniaceae amino acid (protein) sequences (Supplementary Table S3) obtained from the UniProt database (Bateman et al., 2017).

Benchmarking Universal Single-Copy Orthologs (BUSCO) v5.1.2 (Simão et al., 2015) was used to estimate the completeness of the taxonomically filtered *P. lobata* and Symbiodiniaceae transcriptomes. The transcriptome assemblies for *P. lobata* and Symbiodiniaceae were compared against the metazoan and alveolate BUSCO gene sets, respectively. For *de novo* assembled transcriptomes, it is expected that multiple predicted isoforms map to the same BUSCO gene, which can lead to high inferred gene duplication rates. To account for this potential artifact, multiple isoforms of the same gene model that mapped to the same BUSCO gene were counted as a single hit.

### Identification of Symbiodiniaceae clades

RNA sequence reads from each sample (Supplementary Table S2) were mapped against publicly available Symbiodiniaceae transcriptomes (Bayer et al., 2012; Ladner et al., 2012) using a custom Perl script (zooxType3. pl; Manzello et al., 2019). The number of high-quality reads (MAPQ >40) mapped to representative Symbiodiniaceae transcriptome assemblies was used to calculate the proportions of *Symbiodinium*, *Breviolum*, *Cladocopium*, and *Durusdinium* in the sampled *P. lobata* colonies (Manzello et al., 2019). PERMANOVA from the R package, pairwise Adonis (version 0.4.1), was used to test for significant differences in Symbiodiniaceae community composition between sites, zones, and seasons.

### Read abundance estimation

Transcriptomes assembled *de novo* usually generate more gene models and transcripts than predicted from whole-genome sequences, with the read coverage frequently not being uniform across inferred transcripts (*cf.* Hayer et al., 2015). This is often addressed by collapsing inferred transcripts using clustering based on a similarity cutoff chosen prior to differential gene expression analysis. Rather than reducing the size of our transcriptome through transcript clustering based on an arbitrarily chosen cutoff, we followed the “analysis first, aggregation second” approach proposed by Yi et al. (2018), which addresses the issue of uneven read coverage across transcripts by aggregating *p*-values from transcript-level differential expression analysis to identify differentially expressed genes (DEGs). Transcript abundances were estimated using the fast k-mer hashing and pseudo-alignment algorithm implemented in Kallisto v0.46.2 (Pimentel et al., 2017), followed by differential gene expression analysis in R using the Sleuth v0.30.0 (Pimentel et al., 2017) package. Read counts for transcripts belonging to the same gene were aggregated (*p*-value aggregation (Pimentel et al., 2017) to infer differential expression at the gene level.

### Differential gene expression and GO enrichment

Our analysis included several units of analysis to explore the variation in the data: comparisons between sampling sites (and zones of the bay), between the sides of the bay, and seasons. Note that comparisons across seasons include repeated measures of gene expression from the same individuals collected during dry and wet seasons. A likelihood ratio test was used to compare full and null (reduced) models to identify significant DEGs between sites, locations, and seasons while accounting for variation of other factors incorporated in the two models (full model: Site + Season + Side [N or S]; null model: Season + Side). All pairwise comparisons between sites (1 vs. 2, 2 vs. 3, *etc.*) were assessed for DEGs (*q*-value <0.05) with the side of Fouha Bay (N or S) and season used for the null model. Since comparisons 1 vs. 2 and 3 vs. 4 showed extremely low DEGs, sites 1 and 2 were combined into inner zones (closer to the river) and sites 3 and 4 into outer zones (closer to the opening of Fouha Bay) for further comparisons. Wald’s tests were used within Sleuth to obtain log2 fold changes of DEGs between conditions. Genes identified as differentially expressed were separated into up and downregulated (enriched in the inner and outer zones, respectively; Figure 5) and used for GO enrichment analysis using R package GO_MWU using default parameters (Wright et al., 2015). The R packages pheatmap (version 1.0.12) and ggplot2 (version 3.4.2) were used to visualize the heatmap and GO enrichment plots, respectively.

## Results

### Seasonal sedimentation patterns

During the dry season, sedimentation rates showed a gradient from severe sedimentation, exceeding rates of 135 mg cm^-2^ day^-1^ near the river mouth at sites N1 and S1 to moderate rates of 36 mg cm^-2^ day^-1^ at sites N4 and S4 (Figures 1C,D). Comparison of the inner (sites 1 and 2) and outer (sites 3 and 4) zones showed statistically significant differences in sedimentation rates for the dry season (*p*-value = 0.029). The side of the river channel was not significantly different for sedimentation rates (*p*-value = 0.091). During the wet season, sedimentation rates converged, being extremely severe across all sites (*p* = 0.993; Figure 1D). Average sedimentation rates did not differ between seasons for sites N1 and S1, but sedimentation increased for the remaining sites during the wet season compared to the dry season (Figure 1D). These seasonal differences in sedimentation rates were mirrored by differences in rainfall, with precipitation recorded at the Humåtak rain gauge being almost twice as high during the wet season from 21 September 2018 to 22 October 2018 (∼15 mm day^-1^) compared to the dry season from 2 May 2018 to 1 June 2018 (∼8 mm day^-1^) (US Geological Survey monitoring site 131729144393766; USGS, 2023). The only significant difference in salinity and temperature on sampling days was the temperature when comparing between seasons. The maximum temperature difference between seasons on the sampling days at any site was 1.02°C (S4 dry season vs. S4 wet season; *p*-value <0.001). The temperature was very similar for all sites for the 7 days leading up to sampling during both the dry (Figure 1A) and wet (Figure 1B) seasons and not significantly different across sites (wet season *p*-value = 0.123 and dry season *p*-value = 0.549). While salinity varied by site for the 7 days leading up to sampling, the variation in salinity was not significant on the day of sampling for different sites, i.e., <1 and <0.7 PSU for dry (*p*-value = 0.7644) and wet seasons (*p*-value = 0.943), respectively. Salinity comparisons between seasons on the sampling day were not significant (*p*-value = 0.494).

### Species identification, sample selection, and RNA sequencing

**FIGURE 2:**
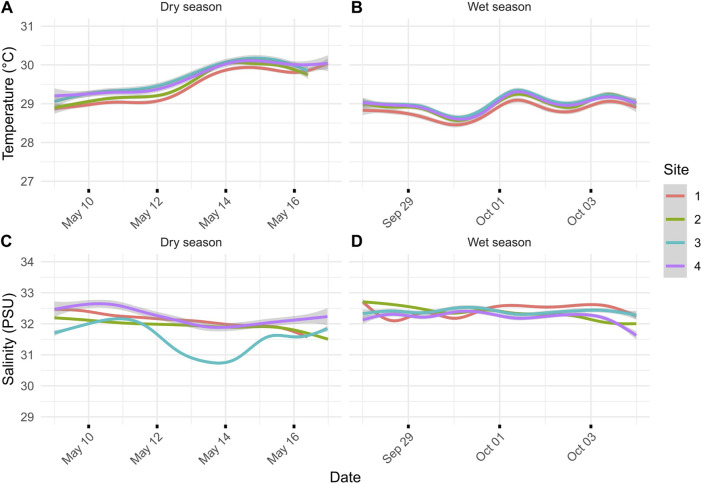
**(A)** Dry-season temperature for the sites during the 7 days leading up to transcriptomic sampling on 16 May 2018. **(B)** Wet-season temperature for the sites during the 7 days leading up to transcriptomic sampling on 4 October 2018. **(C)** Salinity (ppt) during the dry season leading up to transcriptomic sampling. **(D)** Salinity (PSU) during the wet season leading up to transcriptomic sampling. Lines represent the average of two loggers, and gray areas represent 95% confidence intervals: one for the north and south sides of the river channel.

**FIGURE 3:**
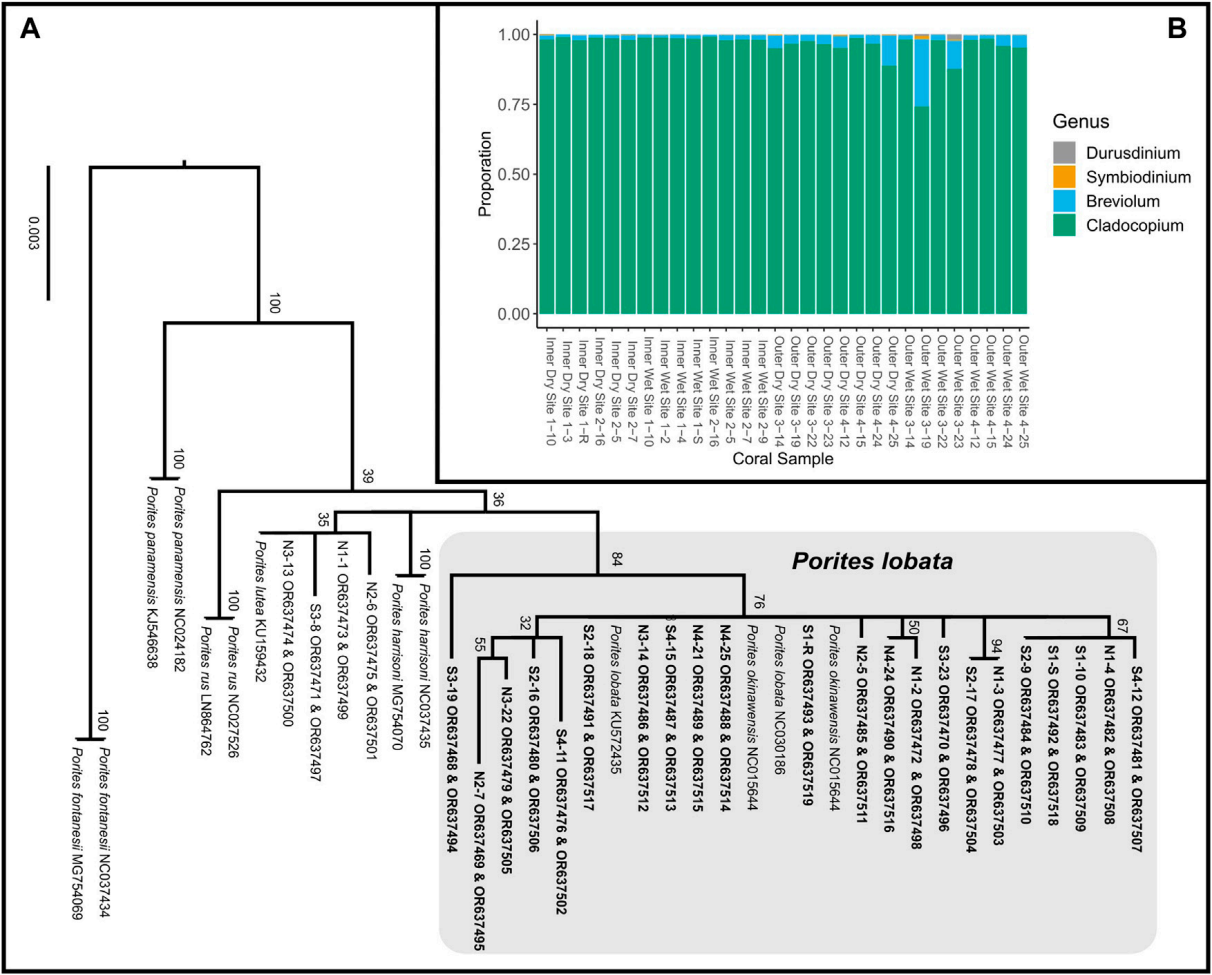
**(A)** Maximum likelihood phylogeny of concatenated mitochondrial COX3-COX2 and ND5-tRNA-Trp-ATP8-COX1 inferred under the GTRCAT model implemented in RAxML (Stamatakis, 2014). Node support was evaluated using 1,000 non-parametric bootstrap replicates. Tagged *Porites* colonies indicated by site, followed by specimen number (e.g., N2-6 represents specimen tag 6 from site N2); colonies in bold were sampled for transcriptome sequencing. ORXXXXXX are the GeneBank accession numbers for that sample. **(B)** Proportionate contributions of Symbiodiniaceae clades contained in each *P. lobata* sample based on read mapping to reference transcriptomes.

Massive *Porites* species are morphologically cryptic (Forsman et al., 2015). Using a DNA barcoding approach, we assigned 20 tagged coral colonies to *P. lobata*, forming a well-supported monophyletic clade, while 4 colonies were assigned to *P. lutea* (Figure 3A) and excluded from further analyses. RNA was extracted from the 20 coral colonies identified as *P. lobata.* For each site (N1 to N4 and S1 to S4; Figure 2C), samples that produced the best-quality RNA extracts (RIN >6) were chosen for sequencing, yielding a total of 30 samples (Supplementary Table S1, 2; Figure 2C) for differential gene expression analysis.

RNA sequencing generated 81,379,001 ± 55,399,881 (mean ± SD) reads, with 73,696,495 ± 51,328,889 reads retained after quality trimming. After *de novo* transcriptome assembly of the filtered reads, removal of contaminants, and taxonomic binning, the resulting *P. lobata* and Symbiodiniaceae transcriptomes contained 72,813 putative gene models (105,623 transcripts) and 57,891 putative gene models (72,428 transcripts), respectively. The transcriptome of *P. lobata* was relatively complete, with almost 90% of BUSCO genes complete (C: 88.5% [S: 78.2%, D: 10.3%], F: 5.0%, M: 6.5%, n: 954); the Symbiodiniaceae transcriptome contained close to 70% of complete BUSCO genes (C: 67.8% [S: 44.4%, D: 23.4%], F: 6.4%, M: 25.8%, n: 171). Relatively higher levels of BUSCO gene duplication are expected in transcriptome assemblies than in genome assemblies, as multiple transcripts (isoforms) may map to the same BUSCO gene. Mapping of Symbiodiniaceae transcripts to publicly available Symbiodiniaceae transcriptomes revealed that all *P. lobata* colonies predominately harbored (>74% relative abundance) *Cladocopium* spp., with most samples showing >95% abundance of *Cladocopium* spp. (Figure 3B; Supplementary Table S4). This is consistent with the findings obtained by Fifer et al. (2022), who showed that *P. lobata*-associated Symbiodiniaceae communities in Fouha Bay were dominated by *Cladocopium* C15. PERMANOVA tests revealed that the site (*p*-value = 0.0750), zone (*p*-value = 0.0518), and season (*p*-value = 0.5649) were not significantly different in Symbiodiniaceae community composition.

### Differential gene expression and GO term enrichment

Likelihood ratio tests of the pairwise comparisons (see *Methods* “Differential gene expression and GO enrichment”) between the sides of the river channel (N vs. S) produced few differentially expressed genes (42 in *P. lobata* and 0 in Symbiodiniaceae*;* Table 2). Comparisons between sites produced similar results for *P. lobata* and Symbiodiniaceae: fewer than two genes were differentially expressed when comparing between sites 1 and 2 and 3 and 4 (Table 1). Given these results, sites were combined into an inner zone (sites 1 and 2) and an outer zone (sites 3 and 4) that correspond to the transition between moderate and severe sedimentation. Taking the variation between zones (inner versus outer) and sides (N or S) into account, the season produced five DEGs for *P. lobata* and none for Symbiodiniaceae (Table 2). Additionally, comparisons of the wet versus dry seasons for only the inner and outer zones revealed between 0 and 11 DEGs for *P. lobata* and none for the Symbiodiniaceae (Table 2). The side of Fouha Bay (north versus south) produced 42 differentially expressed genes for *P. lobata* and none for Symbiodiniaceae.

**TABLE 1:** Number of differentially expressed genes for each pairwise comparison between sites using likelihood ratio model comparisons; factors included in null models given in brackets. Sites were merged into inner (sites 1 and 2) and outer (sites 3 and 4) zones based on the low gene expression between those sites.

*Porites lobata*: inner versus outer zone [side (N/S) + season]					Symbiodiniaceae: inner versus outer zone [side (N/S) + season]				
Site	1	2	3	4	Site	1	2	3	4
1	-				1	-			
2	2	-			2	0	-		
3	736	499	-		3	825	97	-	
4	873	1,333	0	-	4	435	488	0	-

**TABLE 2:** Number of differentially expressed genes identified for *Porites lobata* and its Symbiodiniaceae endosymbionts using likelihood ratio model comparisons; factors included in null models given in brackets. The comparison of inner and outer zones yielded the largest number of differentially expressed genes. Arrows in the last row indicate the number of genes upregulated (↑) and downregulated (↓) in the inner zone, which all subsequent analyses are based on.

Comparison	*Porites lobata*	Symbiodiniaceae
Season: dry versus wet [side (N/S) + zone]	5	0
Season (inner zone): dry versus wet [side (N/S)]	11	0
Season (outer zone): dry versus wet [side (N/S)]	0	0
Side: north (N) versus south (S) [season + site]	42	0
Zone: inner versus outer [season + side (N/S)]	1,702 (↑ 433; ↓ 1,259)	1,514 (↑ 319; ↓1,195)

**FIGURE 4:**
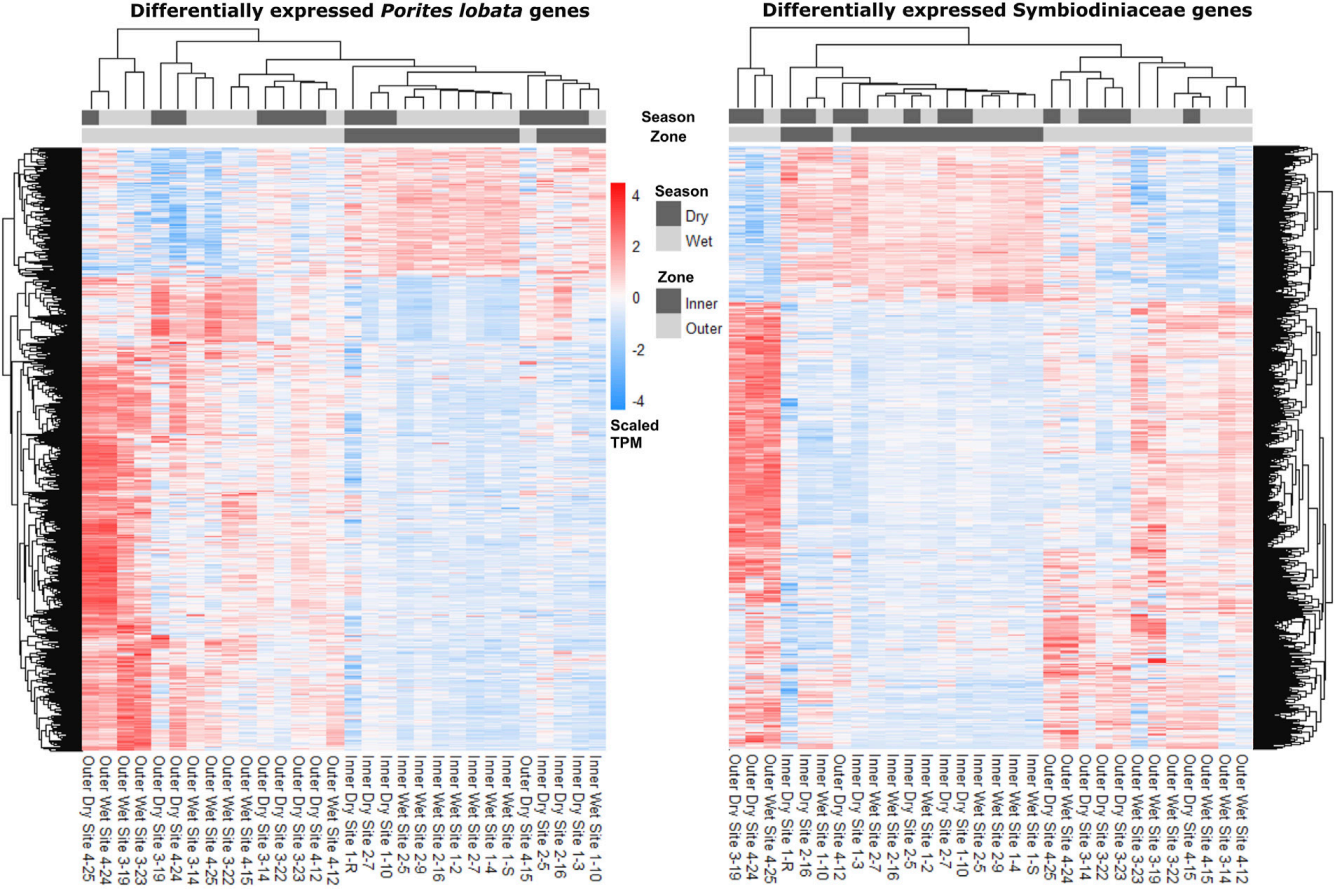
Heatmaps depicting read counts in transcripts per million (TPM) for each differentially expressed gene (rows) for *P. lobata* and Symbiodiniaceae. Samples (columns) are ordered by gene expression similarity. For both *P. lobata* and Symbiodiniaceae, samples cluster by zone rather than season (see *Results* for more information).

**FIGURE 5:**
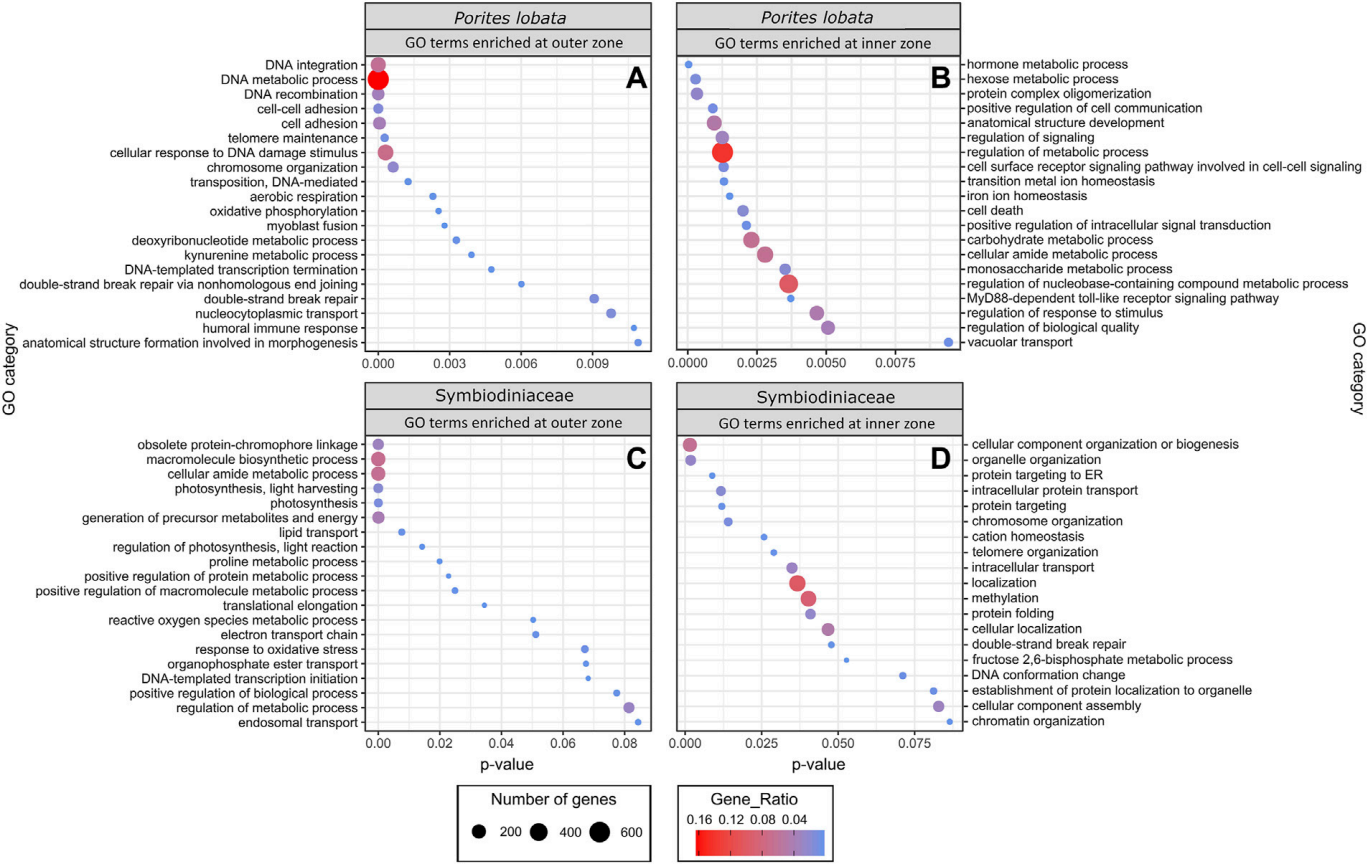
GO terms identified as enriched when comparing inner and outer zones for both *P. lobata* and Symbiodiniaceae. Up and downregulated genes were separated into “enriched in the inner zone” and “enriched in the outer zone.” Gene ratio (color of circles) indicates the number of genes represented by a GO term relative to the total number of differentially expressed genes, whereas the number of genes (size of circle) is the total number in that GO category.

The zone (inner versus outer) produced the largest number of DEGs for both *P. lobata* (1,702 DEGs; 433 upregulated and 1,259 downregulated in the inner zone) and Symbiodiniaceae (1,514 DEGs; 319 upregulated and 1,195 downregulated in the inner zone) (Table 1; Supplementary Table S5). These results mirror the patterns of gene expression profile similarity that largely group samples by zone rather than season, differentiating between the inner zone affected by severe sedimentation and the outer zone impacted by moderate sedimentation (Figure 4). GO enrichment analysis (Supplementary Table S7) identified 38 biological processes enriched (*p* < 0.01) in *P. lobata* (Figures 5A, B) and 10 biological processes enriched (*p* < 0.01) in Symbiodiniaceae (Figures 5C, D), which were separated into GO categories enriched in the inner and outer zones. It is worth noting that gene annotation and subsequent GO enrichment analysis are based on gene homology with mostly vertebrate model organisms that possess immune responses that differ from the innate immune responses of cnidarians. However, we identified the most similar homologs across publicly available cnidarian sequence data that provide our current best understanding of non-model cnidarian genes.

## Discussion

Severe sedimentation in Fouha Bay began in the 1980s because of improper soil management during road construction, which has led to a decrease in coral cover when comparing coral community surveys prior to construction (Randall and Birkeland, 1978) with more recent surveys (Rongo, 2004; Minton et al. 2022). During our sampling periods, sedimentation ranged from moderate (37 mg cm^-2^ day^-1^) to severe (136 mg cm^-2^ day^-1^) during the dry season along the environmental gradient sampled in Fouha Bay (Figure 1D). During the wet season, all sites were inundated with extremely severe levels of sedimentation (>200 mg cm^-2^ day^-1^; range: 199–245 mg cm^-2^ day^-1^) (Figure 1D). Our observations are consistent with those of previous studies that documented and modeled sedimentation rates at times exceeding 200 mg cm^-2^ day^-1^ in Fouha Bay (Randall and Birkeland, 1978; Wolanski et al., 2003; Rongo, 2004; Minton et al., 2022). Rongo (2004) proposed a model of annualized sedimentation rates (N1/S1 = 102.5 mg cm^-2^ day^-1^, N2/S2 = 65.2 mg cm^-2^ day^-1^, N3/S3 = 53.4 mg cm^-2^ day^-1^, and N4/S4 = 22.5 mg cm^-2^ day^-1^), which closely mirrors our sampling period in the dry season and shows a gradient of sedimentation rates decreasing from the river mouth toward the outer parts of Fouha Bay (Figure 1D), with the transition from moderate to severe sedimentation between the inner and outer zones representing a significant difference in sedimentation rates. However, wet season sedimentation documented by us, albeit with limited sampling, showed that the sedimentation gradient in Fouha Bay broke down during the wet season, with all sites becoming impacted by severe sedimentation. Nonetheless, decreases in coral coverage and species richness documented over the last 40 years in Fouha Bay, linked to long-term chronic sedimentation stress, followed the patterns of the dry-season sedimentation gradient, with the lowest coral diversity and abundance near the river mouth, where sedimentation is severe, and coral diversity and abundance increasing as sedimentation decreases to moderate or light levels further from the river mouth (Randall and Birkeland, 1978; Minton et al., 2022). The stark decrease in coral diversity and cover in Fouha Bay linked to the transition from moderate to severe sedimentation has previously been identified as a critical sedimentation threshold (Minton et al., 2022). Sedimentation is the result of a freshwater influx carrying suspended sediments. Considering that freshwater plumes float atop seawater due to their low specific gravity, it is not surprising that salinity at the depths where corals grow in Fouha Bay showed little variation (<1 PSU) across sites (Figure 2). While drastic reductions in salinity (∼10 PSU) have been shown to elicit specific transcriptomic responses (Aguilar et al., 2019), the corals sampled in this study were not exposed to salinity levels significantly below 32–33 PSU, close to the ambient salinity in Guam (34–35 PSU). Given these considerations, the transcriptomic signatures described below are likely and primarily attributable to sedimentation stress.

Despite a large difference in sedimentation rates between dry and wet seasons (Figure 1D), gene expression profiles were more similar across samples within zones than those within seasons (Figure 4; Table 2). Indeed, differential gene expression analysis comparing dry and wet seasons yielded a minimal number of differentially expressed genes in both *P. lobata* and its Symbiodiniaceae endosymbiont community (Table 2). The lack of detectable gene expression differences between seasons may be linked to the chronic nature of the sedimentation stress experienced by *P. lobata* in Fouha Bay, consistent with significant reductions in coral cover and diversity between the outer and inner zones (Minton et al., 2022). The outer zone of Fouha Bay is more exposed to wave action than the inner zone, which would allow for a more expedient dissipation of turbidity and accumulated sediments. Despite this, the larger outer colonies still have significant energetic requirements to remove sediment from their large colonies, especially since the sedimentation rates even for the outer colonies are at the limits of survival for many coral species (Figure 1D; Erftemeijer et al., 2012; Tuttle and Donahue, 2022). Shifts in Symbiodiniaceae communities could theoretically contribute to the mitigation of sedimentation stress, but we found these communities to be largely homogenous and stable (Figure 3B), consistent with the previously reported dominance of *Cladocopium* C15 and lack of spatial structuring of symbiont subclades in *P. lobata* across Fouha Bay (Fifer et al., 2022).

Given that sampling during the wet season occurred following significant rain events, sedimentation rates may have increased temporarily in the outer zone of Fouha Bay, temporarily obscuring the well-described sedimentation gradient of Fouha Bay (Rongo, 2004; Minton et al., 2022). The lack of differentially expressed genes between sites N3/S3 and N4/S4 in the outer zone and N1/S1 and N2/S2 in the inner zone points to an environmental break between zones, consistent with a transition from moderate to severe sedimentation rates (Figure 1D) (Minton et al., 2022). The large number of differentially expressed genes identified when comparing the inner and outer zones using differential gene expression analysis (Table 2), in conjunction with the clustering of inner and outer zones based on gene expression similarities (Figure 4), suggests a strong metabolic threshold associated with the transition from moderate (∼57 mg cm^-2^ day^-1^ during the dry season) to severe (∼85 mg cm^-2^ day^-1^ during the dry season) sedimentation in *P. lobata*. These results are consistent with those obtained by Minton et al. (2022), who identified a critical sedimentation threshold of 48 mg cm^-2^ day^-1^ at which the abundance and diversity of corals significantly decrease in Fouha Bay, with massive *Porites* spp. being one of the few taxa able to persist. Chronic sedimentation affects the metabolism of persisting corals and likely reduces coral recruitment. Limited recruitment and high metabolic costs to survive at the inner site likely led to the observed small, average colony sizes. Furthermore, many of the colonies in this inner zone have perished since the sampling in 2018.

Taken together, coral community survey data (Minton et al., 2022) and our gene expression analysis suggest that *P. lobata* and their Symbiodiniaceae endosymbionts are chronically stressed due to severe sedimentation in the Fouha Bay inner zone. Through comparisons of gene expression patterns (Figure 4; Table 2), annotation of differentially expressed genes, and GO term enrichment analysis (Supplementary Table S3-5), we identified the putative signatures of sedimentation on the energy metabolism and immune response of *P. lobata* and its endosymbiotic Symbiodiniaceae community discussed below. Messenger RNA (mRNA) and protein expression are often poorly correlated (e.g., due to variations in mRNA stability and translational efficiency [Payne, 2015; Liu et al., 2016]), including in corals and their symbionts (Mayfield et al., 2016). While this has led to concerns about interpreting differential gene expression patterns, the correlation of differentially expressed mRNAs with protein expression may be significantly better than for non-differentially expressed genes (Koussounadis et al., 2015). Transcriptomics allows for rapidly profiling tens of thousands of potential biomarkers at once, which we used to provide a framework for the coral sedimentation stress response under field conditions that can guide multi-omics approaches aimed at linking genes to protein expression and their enzymatic and metabolic products to untangle the regulation and complexity of this system.

### Energy metabolism

GO terms associated with oxidative phosphorylation (GO:0006119) and aerobic respiration (GO:0009060) were enriched for *P. lobata* in the outer zone, exposed to moderate sedimentation (Figure 5A). For Symbiodiniaceae, photosynthesis (GO:0015979), generation of precursor metabolites and energy (GO:0006091), electron transport chain (GO:0022900), and lipid transport (GO:0006869) were enriched in the outer zone (Figure 5C). Photosynthesis-associated genes coding for fucoxanthin–chlorophyll a–c binding proteins A, F, B, and E and photosystem I/II reaction center proteins were downregulated in Symbiodiniaceae from the inner zone (Supplementary Table S6), suggesting that the maintenance of the photosynthetic machinery is reduced under severe sedimentation, likely attributable to depleting energy stores (Sheridan et al., 2014), reallocation of energy due to crucial stress responses (Riegl and Branch, 1995), reduced light, and/or decreased oxygen availability (Jones and Hoegh-Guldberg, 2001; Weber et al., 2012). Other studies have reported a significant reduction in the maximum quantum yield (F_v_/F_m_) of photosystem II in response to sedimentation stress from a variety of coral species (Philipp and Fabricius, 2003), consistent with the decreased rates of photosynthesis and a significant reduction in the lipid stores of corals (Sheridan et al., 2014). The upregulation of lipid catabolic and transport enzymes (lipases and apolipoprotein L domain-containing protein 1; Supplementary Table S6) and downregulation of fat storage-inducing transmembrane protein 2 (Supplementary Table S6) in *P. lobata* indicate that corals in the inner zone of Fouha Bay likely deplete their lipid reserves to maintain antioxidant production, an innate immune response, and increased cilia action and mucous production to persist under chronic and severe sedimentation stress that requires the removal of sediment deposits, mitigation of pathogens, and elimination of increasing reactive oxygen species (ROS) that are the result of decreased dissolved oxygen available for metabolic processes in a turbid environment. Reduction in photosynthesis, as suggested by the downregulation of photosystem genes in Symbiodiniaceae observed in this study, may be offset by increased heterotrophic feeding, which has been suggested as an adaptive strategy for corals persisting in turbid environments (Bay et al., 2009; Pacherres et al., 2013), a possible strategy for *P. lobata* that is a mixotroph (Conti-Jerpe et al., 2020). Taken together, our results of GO term enrichment (Figure 5) and differential gene expression (Table 2; Supplementary Table S6) analyses indicate that *P. lobata* in the inner zone showed a decrease in energy generation, likely due to a depletion of energy reserves (Sheridan et al., 2014), and decreased photosynthetic activity of Symbiodiniaceae due to reduced light availability and/or lack of oxygen availability for aerobic respiration (Jones and Hoegh-Guldberg, 2001; Philipp and Fabricius, 2003).

GO terms associated with aerobic energy generation for *P. lobata* were enriched in the outer zone compared to the inner zone (Figure 5A), further indicating that corals in the inner zone experienced hypoxic stress as can be expected under conditions of severe sedimentation (Weber et al., 2012; Bollati et al., 2021). In *ex situ* sedimentation experiments, Bollati et al. (2021) found genes associated with an anaerobic glycolytic pathway upregulated in two coral species. Similarly, we find glyceraldehyde-3-phosphate dehydrogenase (GAPDH) (Supplementary Table S6) upregulated within Symbiodiniaceae in the inner zone and GO terms for aerobic respiration (GO:0009060) and oxidative phosphorylation (GO:0006119) of *P. lobata* enriched in the outer zone (Figure 5A), pointing to a reduction in aerobic respiration in corals of the inner zone to mitigate oxygen limitations caused by severe sedimentation. Furthermore, we found a hypoxia-inducible factor (aryl hydrocarbon receptor nuclear translocator in *P. lobata*; Supplementary Table S6) upregulated in *P. lobata* colonies from the inner zone. While not intuitive at first glance, the observed enrichment of the GO term response to oxidative stress (GO:0006979) and reactive oxygen species metabolic process (GO:0072593) in Symbiodiniaceae of the outer zone (Figure 5C) is consistent with the metabolic differences between inner and outer zones. In particular, increased photosynthetic activity in the less turbid outer zone compared to the inner zone likely elevated photosynthetic rates and increased ROS production in Symbiodiniaceae, which required mitigation. Similarly, antioxidant-associated genes (HSP-70, thioredoxin domain-containing proteins, superoxide dismutase, DnaJ-like proteins, and peroxidase; Supplementary Table S6) were upregulated in *P. lobata* in the outer zone. Our results are consistent with a switch from aerobic to anaerobic-dominated respiration in *P. lobata* from the outer to the inner zone. Quantifying the impacts of such metabolic switching on the health of *P. lobata* using appropriate assays (Murphy and Richmond, 2016) would provide additional insights into the adaptive capacity of this resilient coral species to chronic sedimentation stress and resulting hypoxia.

### Immune response

Corals are known to possess a diverse repertoire of genes involved in innate immunity, with many being unique to *Scleractinia* and specific to certain coral taxa, which helps explain their variable responses to bacterial challenges (Cunning et al., 2018). This includes genes involved in pathogen recognition, signal transduction, and apoptosis pathways. In the marine environment, pathogenic bacteria are often associated with sediment influx and require corals to rapidly respond to these challenges to persist in turbid environments (Weber et al., 2012). In this study, we found GO terms associated with the positive regulation of cell communication (GO:0007267)​​, positive regulation of signal transduction (GO:0009967), MyD88-dependent Toll-like receptor signaling pathway (GO:0002755), cell surface receptor signaling pathway involved in cell–cell signaling (GO:0007166), and regulation of the response to the stimulus (GO:0048583) overrepresented in *P. lobata* growing in the inner zone impacted by severe sedimentation (Figure 5B). The MyD88-dependent Toll-like receptor signaling pathway is involved in the innate immune response of corals to many different stressors (van de Water et al., 2015a; van de Water et al., 2015b; Anderson et al., 2016; Mydlarz et al., 2016; Young et al., 2020), stimulating the expression of pro-inflammatory cytokines (Poole and Weis, 2014). In sponges, this signaling pathway is strongly upregulated in response to bacterial endotoxin lipopolysaccharides, effectively eliminating Gram-negative bacteria through the formation of a recombinant protein (Wiens et al., 2005). Gram-negative pathogenic bacteria, such as *Vibrio* spp., are associated with marine sediments (Franco et al., 2020), and bacterial communities associated with heavy sediment loads are known to exacerbate mortality in corals (Hodgson, 1990; Weber et al., 2012). Indeed, microbiome metabarcoding previously revealed that *P. lobata* colonies growing in the inner zone of Fouha Bay, close to the river mouth, harbored higher abundances of Vibrionaceae than colonies in the outer zone (Fifer et al., 2022).

Although corals are thought to only possess an innate immune system (Palmer and Traylor-Knowles, 2012), the presence of the MyD88-dependent Toll-like receptor signaling pathway is an example of an adaptive-like immune response system that may help corals mitigate bacterial infections (Wiens et al., 2005; Poole and Weis, 2014) associated with severe sedimentation. Only recently, coral immune cells and their gene expression have been characterized using single-cell transcriptomics (Levy et al., 2021). Interestingly, we found genes associated with immune functions in coral immune cells upregulated in the inner zone (interferon regulatory factor 2, tyrosinase, and homeobox genes coding for Meis2 and Nkx-2.2a; Supplementary Table S6). In summary, we observed the enrichment of cell signaling GO terms (Figure 5B) and upregulation of genes previously identified as molecular signatures of the immune function (Palmer and Traylor-Knowles, 2012; Poole and Weis, 2014; Mydlarz et al., 2016; Levy et al., 2021) in *P. lobata* colonies growing in the inner, severely sedimentation-impacted zone, suggesting that coral colonies in the inner zone rely on their immune response to mitigate the impacts of severe sedimentation.

Despite environmental sensing and resulting metabolic and immune responses, cell damage under severe stress is expected to increase. The initiation of apoptosis is a critical process that allows organisms to maintain tissue homeostasis by eliminating damaged cells that, for example, pose a risk for infection by pathogens (Ameisen, 2002). Various programmed cell death pathways have been identified in the cnidarian stress response, but the destabilization of cellular adhesion seems to be a crucial component in apoptosis initiation, given that changes in cell adhesion proteins have been associated with the response to coral bleaching (Gates et al., 1992; DeSalvo et al., 2012; Traylor-Knowles and Connelly, 2017), disease (Daniels et al., 2015), and heat plus sedimentation stress (Poquita-Du et al., 2019). Consistent with these prior studies, we found GO terms for cell death (GO:0008219) enriched in the inner zone (Figure 5B), as well as the enrichment of cell adhesion terms (GO:0007155, GO:0098609) in the outer zone (Figure 5A). This pattern suggests that *P. lobata* from the inner zone modified the extracellular matrix of part of its population of cells in conjunction with elevated rates of apoptosis initiation to remove cells damaged by severe sedimentation stress in an attempt to survive in this turbid, marginal habitat.

## Conclusion

Transcriptomics provide a valuable insight into the rapid physiological response of an organism, but differential gene expression analysis may fail to capture longer-term responses such as seasonal differences, which may be more appropriately investigated with methods such as epigenomics. Nevertheless, in this study, we sampled corals along an established sedimentation gradient and provide a framework of the metabolic acclimation response of *P. lobata* colonies and its Symbiodiniaceae endosymbionts to varying sedimentation rates under *in situ* field conditions.

We identified processes that may allow for sedimentation-tolerant corals, such as *P. lobata*, to persist in habitats impacted by chronic and severe sedimentation. Switching between energy generation pathways may help corals living in sedimentation-impacted, turbid reefs maintain a stress response to survive when the availability of light and oxygen is diminished. Rapid environmental sensing and cell–cell communication allow corals to respond to freshwater influx, sedimentation stress, and bacterial challenges associated with terrestrial runoff. Given the cellular damage associated with environmental stress, the removal of damaged cells through programmed cell death pathways is crucial to maintaining coral colony integrity and closing entryways to pathogens. While many of these implicated pathways are involved in a range of coral stress responses, from bleaching to contamination from anthropogenic chemicals, here we observe these responses of *P. lobata* colonies to increasing sedimentation along a gradient in Fouha Bay, Guam. These putative mechanisms identified by us using transcriptomics ought to be further investigated and tested using additional approaches, in particular those that capture persistent and heritable modifications of traits under field and controlled conditions, to gain a better understanding of sedimentation stress-tolerance mechanisms in corals. Coastal development activities continuously increase sedimentation and turbidity in near-shore reefs, and a more in-depth understanding of the metabolic plasticity of diverse corals will facilitate predicting the acclimation and adaptation limits of near-shore reef ecosystems in the Anthropocene.

## Data Availability

The datasets presented in this study can be found in online repositories. The names of the repository/repositories and accession number(s) can be found in the article/Supplementary Material.
